# Carbolic Acid and Creasote—Their Chemistry and Therapeutical Application to the Practice of Dentistry

**Published:** 1880-12

**Authors:** Truman W. Brophy

**Affiliations:** Chicago.


### ARTICLE II.

### *Carbolic Acid and Creasote—Their Chemistry and Therapeutical Application to the Practice of Dentistry.*

BY TRUMAN W. BROPHY, M. D., D. D. S., CHICAGO.

There are no remedies mentioned in the Materia Medica, so extensively used in dentistry, as the ones I have selected as the subject of this paper. Therefore, I believe, we can occupy our time in no better way than to examine their merits, as well as to consider where they are indicated, and how they should be applied. Let us first look into the chemical composition of these substances and then speak of their medicinal properties. Carbolic Acid has for its formula, H 2 C 6 H 4 O. Carbolic acid is known by the names of hydrate of phenol, C 6 H 5 A O, phenic acid, phenic alcohol and phenol. It is only slightly soluble in water, forming *aqua acidi carbolici* of the U. S. P., but it dissolves readily in alcohol, ether and glycerin. When liquefied by heat, or by five per cent. of water, it resembles creasote, and is very frequently substituted for it.

Coal tar oil, besides furnishes carbolic acid, or hydrate of phenol, contains cresyl, hydrate of cresyl, or crsylic acid C 7 H 7 H O. While wood-tar oil contains guiacal, C 7

H 8 O 2, also C 8 H 10 O 2, or the creasote with which we are all so familiar. Carbolic acid may be distinguished from creasote by the following tests: Carbolic acid boils only at 370° F., while creasote dries up at 212°. Carbolic acid is either solid, or may be rendered so by subjecting it to cold; creasote cannot be solidified by the cold produced by a mixture of hydrochloric acid and sulphate of sodium. Carbolic acid is soluble in a large volume of water (about 1 in 80 or 1 in 20, of boiling water.) This solution is colored blue by a neutral solution of feric chloride, while wood creasote is less soluble in water (1 in 129) and has not a blue color imparted to it by feric chlorid. An alcoholic solution of carbolic acid is colored brown by feric chlorid; a similar solution of wood creasote, green. Carbolic acid forms a jelly when shaken with collodion; not so with creasote. There has been considerable discussion on the subject as to whether carbolic acid is a *true* acid. While it does not color blue litmus paper, red, this failing to respond to the usual test for acids, we know that it unites with basylous radicals, forming carbolates, therefore it is an acid.

There is much in the literature of dentistry on the use of carbolic acid and creasote; there seems to be an almost universal preference shown for the latter. Carbolic acid has entirely superseded creasote as a remedy in general medicine and surgery, and I believe there is no sufficient reason for placing it second to creasote in dental therapeutics. Why creasote is preferred to carbolic acid by dentists, is indeed difficult to understand. A disposition on the part of members of the profession, however, to accept the method of their predecessors, may be an explanation of the views held by so many in regard to these agents. Since carbolic acid is equal and perhaps superior to creasote, as a therapeutical agent, I shall speak of it hereafter, and shall insist that its action is identical to that of creasote when of the same strength, and their strength is determined by their power to coagulate albumen. Of twentyfive scruples of creasote purchased from as many dealers, including dealers in dental goods, I find on examination that all, with but one exception, contains carbolic acid—the one which is free from carbolic acid was imported and sold to me by Gale & Blocki, of Chiago. Those, therefore, who supposed they were using creasote have employed either carbolic acid or a mixture of this acid with creasote. Carbolic acid is soluble in water only in proportion of ninety-five per cent. of carbolic acid to five per cent. of water, and in any proportions stronger than that; also in the proportion of ninety-five per cent. of water and five per cent. of carbolic acid or any solutions weaker than that. It is impossible to dissolve carbolic acid in water alone, in any other proportions than the above. If, however, we add glycerine or alcohol to the water we may make it of any strength desired. We hear of ten per cent. solutions of carbolic acid and water—this is an impossibility.

I cannot illustrate the views held by me in regard to the use of remedies more forcibly than by quoting the words of a distinguished physician, and one whom you will recognize as reliable authority on all matters pertaining to medicine. I refer to Prof. J. Adams Allen, who in his open lecture on general therapeutics, at Rush Medical College, said: "We must trace out an essential relation between our therapeutical agents and the changes produced by disease, in order that we may intelligently apply the former to the latter. But if we cannot establish this relation, we must throw our agents aside as useless. Because Drs. A, B and C have used a certain remedy in certain diseases, a certain number of times, and their patients have recovered, it is not proof that it was the result of using that particular agent. We must prove that there is a relation between the remedy we use and the disease for which we employ it. *Disease* is change of structure of a part from a normal standard. A remedy is an agent which restores the part to its normal condition, no matter

*how* it does it, so it is *done*, but in the first place we must ask a question: *What's the matter?* What material change has taken place, how can we restore it to its normal standard? How can we bring it back? Anything that will do that *is a remedy*, if it does not, it is *not*. If it aggravates the conditions existing, it is a *poison*, that's all."

There is sufficient in the foregoing remarks to guide us, keep us in the right path, and prevent us from misapplying remedies the properties of which are so clearly set forth in our works on therapeutics. Is carbolic acid so used, especially when applied to exposed pulps, that it will restore them to their *normal standard?* Let us see. In the first place it is essential to know the action of this agent when locally applied. Carbolic acid coagulates albumen, and dissolves into volatile and fixed oils. It is an escharotic, leaving a white eschar which soon turns brown and is cast off. Carbolic acid is an anæsthetic, acting with wonderful promptness upon the part to which it is applied.

Dr. J. H. Bill states in an article published in the *American Journal of Medical Science*, that "by applying carbolic acid to the integument, insensibility to pain may be sufficiently induced to permit free incisons without suffering." The escharotic action of carbolic acid contraindicates its use as an application to exposed pulps, the vitality of which we endeavor to preserve. If, however, we make a solution from one to five per cent. we have an excellent remedy, slightly stimulating and antiseptic, which may be applied to an exposed pulp without fear of causing a slough and subsequent death of the whole part. I desire, gentlemen, to repeat what I stated to you three years ago, to wit: That I regard it bad practice to apply an escharoitc to a pulp, the vitality of which may be preserved by proper treatment. Carbolic acid or creasote, therefore, if used in contact with exposed pulps, which we subsequently cap, must not be stronger than a five per cent. solution. Carbolic acid has been very successfully used to arrest nausea and vomiting. From one to two drops in a glass of water will usually accomplish the result desired.

Since carbolic acid has the power to arrest suppuration, and to promote healthy granulations, it is invaluable in the treatment of alveola abcesses, apthæ, ulitie, stomatitis, phagedæna, papilloma, epithelioma, schirrous, cauliflower growths, etc. In the treatment of the above dis eases, the agent should be of such strength as the requirements of the case indicate.

Carbolic acid *is a powerful* antiseptic, but *is not*, as many well-informed members of the profession believe, a disinfectant. Carbolic acid *has* the power of preventing decomposition (hence *it is* an antiseptic), but *has not* the power to destroy or render inoffensive the products of decomposition; therefore it *is not* a disinfectant.

Owing to the erroneous impression as to the properties of carbolic acid, it is used to the detriment of patient and practitioner in the treatment of many lesions. Carbolic acid is an irritant poison. Although an escharotic it diffuses into the blood rapidly. If carbolic acid has been taken in toxic doses, liquor calcis, saleratus or oil is the antidote. When it is found necessary to devitalize a pulp, the anæsthetizing properties of undiluted carbolic acid make it desirable as a dressing previous to the application of arsenious acid. There are other remedies, however, equally as efficient. Carbolic acid is the most satisfactory agent to obtund sensitive dentine without endangering the vitality of the pulp, with which I am acquainted. Carbolic acid may very properly be applied to the canals of teeth immediately after extirpating their pulps, but if the pulp is putrescent it is not to be used; since some form of chlorine is the agent indicated with which to disinfect the parts.

Carbolic acid as well as creasote, are flesh preservers; indeed, the word creasote is derived from two Greek words, *Creas*, flesh, and *Sotal*, I preserve—flesh preserver. It preserves flesh, however, not as is frequently supposed, to wit: by preventing vital soft parts from losing their vitality, but by preventing non-vital tissue from becoming putrid and disintegrating. Carbolic acid is a most excellent remedy to apply to carious teeth during the process of excavating, to enable us to more clearly see the extent of the caries. In many cases where we have adjusted the rubber cloth and excavated the cavity in the most skillful manner, we are suprised, upon moistening the cavity and its surrounding surfaces with carbolic acid, to find carious tissue, which if permitted to remain, would soon render another operation necessary, in arresting the progress of disease. If a few drops of carbolic acid be added to sandarac varnish, which we use for moistening cotton for the temporary sealing of cavities, it will prevent decomposition of the secretions which accumulate about the cavity and the cotton will remain free from offensive odors much longer than it otherwise would. Carbolic acid is most destructive to the lower forms of life. These minute organisms so frequently found in the oral cavity and in carious teeth cease to exist when very dilute solutions of this agent are brought in contact with them! "As all fermentations are correlative of the growth and multiplication of these minute bodies, carbolic acid, by destroying their activity, arrests zymosis." A solution, one per cent. in strength, according to Dr. Isador Neumann, is sufficient to destroy bacteria, vibrio, etc. Much more might be said in regard to the use of carbolic acid in dentistry, but I shall detain you no longer. I hope there may be untiring research without prejudice, by the members of our profession, as to the action of remedies, so that we may soon say that empiricism has yielded to the higher, more reliable and satisfactory methods, based upon the grand, broad, beautiful and universal truths revealed to us through the instrumentality of science.

After reading his paper Dr. Brophy exhibited the collodion test for distinguishing carbolic acid from creasote.

DISCUSSION.

Dr. Cushing:—I have procured an article of colorless creasote from Sargent, imported by him which he believes to be pure.

Dr. K. B. Davis:—I have found carbolic acid to be much more escharotic than creasote.

Dr. Brophy:—I agree to that, but claim that creasote has no properties not possessed by carbolic acid of the same strength.

Dr. Spalding:—What is the standard of strength since creasote and a 95 per cent. solution of carbolic acid are not of equal strength? The experiment of coagulating collodion is merely for identifying them, and is not a test of relative strength.

Dr. Kulp:—What are the comparative merits of the two for the treatment of abscess as shone by experience? That may perhaps throw as much light upon the question of their relative strength as a theoretical examination.

Dr. Harlan:—I wish to correct one error in the paper. Carbolic acid in full strength is not an anæsthetic. That property is only developed by dilution and evaporation. Pure wood creasote reddens litmus paper and does not coagulate albumen to anything like the same extent as carbolic acid, and is much inferior to carbolic acid for use in practice.

Dr. Brophy:—A 95 per cent. solution of carbolic acid is anæsthetic.

Dr. Hurtt:—I have often verified the statement of the paper as to the anæsthetic properties of carbolic acid by using it for that purpose in making incisions into abscesses.

Dr. Talbot:—The same thing is shown in the old method of capping exposed pulps by bathing them with carbolic acid or creasote and covering with oxychloride of zinc. The severe pain which the contact of the chloride of zinc would otherwise cause, being prevented by the anæsthetic effect of the carbolic acid.

Dr. Crouse:—The chloride of zinc does not come in actual contact with the pulp on account of the coagulum or eschar between, which was made by the carbolic acid, and I think that accounts for the absence of pain rather than any anæsthetic effect of the carbolic acid.

Dr. Talbot:—The chloride of zinc would penetrate the coagulum and still produce pain except for the anæsthetic effect of the carbolic acid.

Dr. Black:—I do not believe that chloride of zinc will penetrate a coagulum, but in many cases an application of creasote or carbolic acid does not reach actual contact with the pulp. If it can be made to do so, and forms a *complete* eschar over the entire exposed surface of the pulp, there will then be no pain from the chloride of zinc, but that is often a very difficult thing to do.

Dr. Hurtt:—There are many young men in the state who have been led into difficulty by the statements made in the society, in former years, about capping pulps with oxychloride of zinc without pain—*without* the warning that Dr. Black has given of the difficulty there is in making the preliminary application of carbolic acid complete and effectual. In one instance I filled a cavity in which there was an exposed pulp (in grinding surface cavity in lower molar,) full to the brim with carbolic acid and left it there for fifteen minutes, and then capped it with oxychloride of zinc without wiping away quite all of the carbolic acid; but after ten or fifteen minutes there was excruciating pain, which required two hours to relieve, after removing the oxychloride of zinc, and which the patient remembers with ill feeling toward me to this day.

Dr. Harlan:—When the pain in an exposed and inflamed pulp is relieved by an application of creasote or carbolic acid, it is not the anæsthetic effect of the application that stops the pain, but it is the exclusion of the air and other irritants from contact with the pulp by the eschar which the applicatian produces, and anything that can get through the eschar may produce pain.

Dr. Brophy:—Will *any* escharotic have the same effect in quieting the pain?

Dr. Harlan:—Some escharotics will not act in that way, but will increase the pain and irritation.

Dr. Brophy :—I insist upon it, that it is the anæsthetic effect of the carbolic acid application which relieves the pain in these cases.

Dr. Taylor :—How long will the anæsthetic effect of the application last ? I believe that the carbolic acid has some effect upon the circulation which prolongs the relief from pain.

Dr. Black :—What has been said about the difficulty of obtaining a *complete* eschar would lead any one to be careful, and not be surprised if there were pain in some cases.

Dr. Hurtt:—Pain will ordinarily be prevented if the slab on which the oxychloride is mixed be warmed, and the material mixed as thick as it can be handled without using pressure upon the pulp.

Dr. Harrison asked Dr. Black the difference between carbolic acid and creasote for the treatment of abcesses.

Dr. Black :—I think them alike—if I have had any creasote that was free enough from mixture with carbolic acid, to afford me a fair comparison.

Dr. Brophy called attention to the statement that an escharotic should never be applied in direct contact to an exposed pulp.

Dr. Black :—I made the same assertion at the previous meeting of this society in Bloomington (ten years ago), and I still believe so. In a paper at that time I gave a record of ten years of indiscriminate capping (with oxychloride of zinc,) and followed by general failures (as was characteristic of the general practice of that time,) but I believed then and now that capping with oxychloride of zinc is good practice in *selected* cases.

Dr. Sturgiss thanked Dr. Brophy for his efforts and investigations in regard to the subject.—*Trans. Ill. State Dental Society.*

